# A2 ACUTE COLITIS CAUSES CHANGES TO THE EFFICACY OF CANNABINOID-1 AND MU-OPIOID RECEPTOR AGONISTS ON INHIBITING ABDOMINAL PAIN

**DOI:** 10.1093/jcag/gwac036.002

**Published:** 2023-03-07

**Authors:** Q K Tsang, C E Degro, H M Schincariol, A E Lomax, S J Vanner, S J Vanner, D E Reed

**Affiliations:** Gastrointestinal Diseases Research Unit, Queen's University, Kingston, Canada

## Abstract

**Background:**

Abdominal pain is a debilitating symptom in patients with inflammatory bowel disease. Previously we have shown that both cannabinoid-1 receptor (CB1R) and mu opioid receptor (MOR) agonists inhibit mechanosensitivity of colonic nociceptive nerves in healthy mice. However, it is unknown whether CBR and MOR agonists continue to have effects during colitis.

**Purpose:**

To determine the effects of CBR and MOR agonists on colonic nociceptive nerves during acute colitis.

**Method:**

Colitis was induced in male and female C57BL/6 mice using 2.5% dextran sodium sulfate in drinking water. Visceromotor response (VMR) to colorectal distention (CRD) (volume range 20-80 µL) was measured using telemetric transmitters. Mice were injected intraperitoneally with vehicle, ACEA, a selective CB1R agonist and/or morphine, a MOR agonist, 30-minutes prior to distention. Extracellular afferent nerve recordings were obtained from *ex vivo* flat sheet preparations of mouse distal colon. Mechanosensitivity of single afferent axons was assessed via mechanical probing of the colon with a 1g von Frey hair before and after superfusion of ACEA and/or DAMGO (MOR agonist). Data were analyzed using a one- or two-way ANOVA with Bonferroni test. N denotes number of mice; n denotes number of single afferent axons.

**Result(s):**

ACEA (3 mg/kg), a dose that significantly inhibited VMR in healthy mice, did not inhibit VMR in mice with colitis (p=0.55, N=6). At a dose that previously had no effect in healthy controls, morphine (0.3 mg/kg) significantly inhibited VMR to CRD, when compared to vehicle (34% reduction, p<0.01, N = 8). A combination of a sub-analgesic dose of ACEA (0.3 mg/kg) with morphine (0.3 mg/kg) significantly reduced VMR (p<0.01, N=5); at 60 µL and 80 µL distention there was a 44% and 49% reduction, respectively (p<0.05 for both). Interestingly, the effect of the combination of ACEA and morphine was larger than that of morphine alone (0.3 mg/kg), but this did not reach statistical significance (-62% vs. -34%; p=0.06, N=5-8). In extracellular afferent nerve recordings, compared to the previous findings in healthy mice, a higher concentration of ACEA (10 µM) was required to inhibit mechanosensitivity (15.2 vs. 11.6 Hz; p<0.05, n=11, N=6) whereas 100nM (p>0.99, n=7, N=5) and 1µM (p=0.25, n=10, N=6) had no effect. DAMGO (1 nM), which previously had no effect in healthy controls, had a tendency to reduce colonic mechanosensitivity, but this did not reach statistical significance (p=0.12, n=7 units, N=5). Interestingly, a combination of sub-analgesic concentrations of ACEA (100 nM) and DAMGO (1 nM) significantly reduced colonic mechanosensitivity (18.4 vs. 12.0 Hz; p<0.05, n=7, N=5).

**Conclusion(s):**

At doses that previously inhibited nociception in healthy mice, CB1R agonists may have lost their analgesic effect during acute colitis. Conversely, less of MOR agonists may be needed to achieve analgesia. Interestingly, sub-analgesic doses of CB1R agonists potentiate the analgesic effect of the MOR agonist during colitis.

**Please acknowledge all funding agencies by checking the applicable boxes below:**

NRC

**Disclosure of Interest:**

None Declared

